# Fluorescent Nanoparticles Synthesized from DNA, RNA, and Nucleotides

**DOI:** 10.3390/nano11092265

**Published:** 2021-08-31

**Authors:** Maofei Wang, Masaki Tsukamoto, Vladimir G. Sergeyev, Anatoly Zinchenko

**Affiliations:** 1 Graduate School of Environmental Studies, Nagoya University, Furo-cho, Chikusa-ku, Nagoya 464-8601, Japan; wang.maofei@b.mbox.nagoya-u.ac.jp; 2 Graduate School of Informatics, Nagoya University, Furo-cho, Chikusa-ku, Nagoya 464-8601, Japan; tsukamoto@i.nagoya-u.ac.jp; 3 Department of Chemistry, M.V. Lomonosov Moscow State University, 119899 Moscow, Russia; sergeyevvg@gmail.com

**Keywords:** DNA, RNA, nucleotides, hydrothermal synthesis, nanoparticles, fluorescent nanomaterial, biodots

## Abstract

Ubiquitous on Earth, DNA and other nucleic acids are being increasingly considered as promising biomass resources. Due to their unique chemical structure, which is different from that of more common carbohydrate biomass polymers, materials based on nucleic acids may exhibit new, attractive characteristics. In this study, fluorescent nanoparticles (biodots) were prepared by a hydrothermal (HT) method from various nucleic acids (DNA, RNA, nucleotides, and nucleosides) to establish the relationship between the structure of precursors and fluorescent properties of biodots and to optimize conditions for preparation of the most fluorescent product. HT treatment of nucleic acids results in decomposition of sugar moieties and depurination/depyrimidation of nucleobases, while their consequent condensation and polymerization gives fluorescent nanoparticles. Fluorescent properties of DNA and RNA biodots are drastically different from biodots synthesized from individual nucleotides. In particular, biodots synthesized from purine-containing nucleotides or nucleosides show up to 50-fold higher fluorescence compared to analogous pyrimidine-derived biodots. The polymeric nature of a precursor disfavors formation of a bright fluorescent product. The reported effect of the structure of the nucleic acid precursor on the fluorescence properties of biodots should help designing and synthesizing brighter fluorescent nanomaterials with broader specification for bioimaging, sensing, and other applications.

## 1. Introduction

Preparation of functional materials from various types of biomasses has attracted considerable attention during past decades [1,2]. Among available biomass feedstocks, nucleic acids (e.g., DNA, RNA) represent ubiquitous biomacromolecules readily available at a reasonable cost from fish milt, plants, yeasts, and other renewable sources. While the occurrence of nucleic acids is lower compared to polysaccharide types of biomass, and the adaptation of DNA for materials production is still in progress, potential applications of DNA in biomedical [3,4,5], environmental [6,7,8,9,10], electronic [11], optical [12], and engineering [13,14,15] fields have been repeatedly demonstrated. A very recent report introduced the concept of “DNA biomass” highlighting transformation of DNA to functional soft materials and even to biodegradable plastics [13].

Among different scenarios of biomass-to-materials conversion, biomass carbonization by hydrothermal (HT) treatment and pyrolysis has been vigorously investigated [1,16,17,18] for sustainable production of fuels [19], chemicals [20], adsorbents [21], fluorescent probes [1,22], and other key materials. In particular, carbon dots (CD) produced from biomasses [23,24] are inexpensive compared to the conventional fluorescent dyes, have high fluorescence [25], and low toxicity [26] that renders them suitable for biomedical [27], energy [28], and catalytic [29] applications. Comprehensive studies can be found on CDs synthesized from cellulose [30], lignin [31], starch [32], chitosan [33], carrageenan [34], and other types of biomass [35]. CDs obtained from pure biomass polymers have a maximum excitation around *λ* = 350 nm and maximum emission at *λ* = 400–450 nm, which is similar to other carbon dots prepared from synthetic precursors [36]. Direct conversion of biomass waste, such as waste fruits and vegetables, waste kitchen garbage, and so forth yields multicolour CD products with a great variation in fluorescence emission wavelength [37,38].

In contrast to carbohydrate-derived CDs, only a few studies have attempted to synthesize fluorescent nanomaterials from DNA by the HT method so far [39,40,41,42]. Ding et al. successfully prepared and applied DNA-derived CDs for simultaneous DNA delivery and visualization [39]. DNA CDs were ca. 6 nm in diameter and had the maximum emission intensity at *λ* = 450 nm under excitation at *λ* = 370 nm. Guo et al. used salmon sperm DNA to prepare fluorescent dots at low temperatures (80 to 180 °C) [40] and applied them for cell imaging. By comparison of CDs prepared from DNA and those from individual DNA bases and base pairs, it was noted that only cytosine bases or cytosine-containing base pairs could be converted to CDs with a bright fluorescence comparable to CDs from DNA. Pandey et al. used salmon sperm DNA to prepare multifunctional CDs of 4–5 nm size for bioimaging, drug delivery by CDs hydrogel, and biosensing [41]. At present, however, to the best of our knowledge, only the four above studies are available and the process of fluorescent dots formation from DNA and factors that control fluorescent properties of CDs are still poorly understood. Furthermore, no literature can be found about HT transformation of RNA, a close analogue of DNA, that can also be obtained from natural sources. Unlike many of carbohydrate precursors having relatively simple chemical structure, DNA and RNA are heteropolymers composed of four types of nucleotides, respectively. In turn, monomers contain sugar, heteroaromatic nucleobases, and phosphate groups and each of these structural units may potentially affect CD characteristics. However, while preparation of fluorescent materials from individual nucleotides can be found in the literature [43,44,45], the correlation of their structures and fluorescent properties was not discussed, nor were the properties of CDs from monomeric and polymeric precursors accurately investigated and compared.

Herein, we successfully synthesized and systematically studied the effect of a nucleic acid structure and synthetic conditions of HT treatment on fluorescent properties of fluorescent nanoparticles (biodots) prepared by HT treatment of DNA, RNA, nucleotides, and nucleosides in the temperature range of 150–250 °C. Biodots prepared from AMP and GMP nucleotides showed superior fluorescence properties and quantum yields in comparison to the products of HT treatment other natural compounds such as amino acids and saccharides. Due to their natural origin, biodots can be applied for biomedical applications: bioimaging, drug delivery, and the analysis of body fluids. By using recognition properties of nucleic acids toward metal ions, nucleic acid biodots can also be used for detection of environmental pollution with heavy metals. Here, for the first time, we revealed that the synthesized biodots showed a marked difference of fluorescent characteristics which should help in designing and synthesizing brighter, more sensitive, and more selective fluorescent nanoparticles for applications in the above fields.

## 2. Materials and Methods

### 2.1. Materials

Deoxyribonucleic acid from salmon milt (ca. 100–300 bp, purity over 90%) was purchased from Fujifilm Wako (Osaka, Japan). DNA sodium salt (ca. 20,000 bp) from salmon milt was a gift of Maruha Nichiro Holdings (Tokyo, Japan). RNA from yeast and guanosine (98%) were purchased from Tokyo Chemical Industry (Tokyo, Japan) and D-(−)-ribose (99.0%) was purchased from Wako Fujifilm (Japan). Cytidine-5′-monophosphate (purity 98%), adenosine-5′-monophosphate (purity 98%), guanosine-5′-monophosphate disodium salt (purity 97%), uridine-5′-monophosphate disodium salt (purity 98%), cytidine (98%), and thymidine (98%) were purchased from Combi Blocks Inc. (San Diego, CA, USA).

### 2.2. Methods

*UV-vis spectroscopy.* UV-vis spectra of nucleic acids and biodots in Milli-Q water were recorded on a V-630 Bio spectrometer (Jasco, Japan) in 1 cm × 1 cm × 5 cm quartz cells (optical path 1 cm) at a room temperature.

*Fluorescence spectroscopy (FS).* Fluorescence spectra of carbon dots in Milli-Q water were recorded on an FP-6600 spectrofluorimeter (Jasco, Japan) in 1 cm × 1 cm × 5 cm quartz cells (optical path 1 cm) at room temperature at 275–400 nm excitation wavelengths.

*FTIR spectroscopy.* Fourier transform infrared spectra of DNA and freeze-dried biodots were recorded on a FTIR-460 spectrometer (Jasco, Japan) at room temperature. A powder of a sample was placed between KBr thin plates and compressed to form a thin disc. Finally, the samples were scanned in a wavenumber range between 4000 cm^−1^ and 400 cm^−1^ and 16 scans were averaged at a resolution of 4 cm^−1^.

*Thin layer chromatography (TLC).* TLC analysis was performed using silica gel 60 on aluminium sheet plates (Merck KGaA, Germany) and BuOH:Acetic Acid:H_2_O = 4:1:2 (by volume) mixed solvent as an eluent.

*Nuclear magnetic resonance spectroscopy (NMR).* The D_2_O solution (0.67 mL) of DNA HT treatment product (ca. 3 mg) was transferred into a 5 mm NMR tube and ^1^H and ^31^P NMR spectra were measured on a JNM-ECA500 instrument (JEOL, Tokyo, Japan). After the measurements, 10 μL of CH_3_CN–D_2_O (10% *v*/*v*) was added to the above NMR tube and ^13^C NMR spectrum was measured. Chemical shifts of ^1^H, ^31^P, and ^13^C NMR in D_2_O are expressed in parts per million (ppm) relative to HDO at δ 4.79 (at 23.9 °C), [46] external 85% H_3_PO_4_ at δ 0.00, and a trace amount of CH_3_CN at δ 1.47, [46] respectively: ^31^P NMR (202 MHz) δ 1.70; ^13^C NMR (125 MHz) δ 11.9, 42.0, 101.7, 110.7, 139.7, 144.1, 153.1, 168.3, 171.7.

*Transmission electron microscopy (TEM).* TEM observations were performed using a JEM-2100 Plus microscope (JEOL, Japan) at 200 kV acceleration voltage. A drop of a solution containing biodots (as prepared) or a suspension of residual precipitate was placed onto carbon film-coated TEM grids (Alliance Biosystems, Japan). The solution was removed after 5 min with filter paper, and the grids were dried in a dry box overnight at relative humidity < 10% before observation.

*Elemental analysis.* Elemental analyses were performed on a Yanaco MT-6 analyser.

### 2.3. Sample’s Preparation

Sodium salts of nucleic acids and nucleotides were dissolved in Millli-Q water to prepare 1% (*w*/*v*) solutions. Nucleotides received as acids were dissolved in water containing an equimolar concentration of NaOH. Nucleosides, due to their limited solubility in water at room temperature, were processed as 1% (*w*/*v*) dispersions. Ten mL of 1% (*w*/*v*) solution of a nucleic acid precursor was transferred to a 25 mL PTFE lined HT autoclave reactor vessel HU-25 (SAN-AI Kagaku, Japan), and the HT treatment was performed at temperatures 150–250 °C and various times. After HT treatment, the reaction mixture was chilled to ambient temperature and a precipitate (if present) was separated by centrifugation at 4000 rpm for 10 min. To remove low-molecular-weight products, the resulting solution was dialyzed against 500 mL Milli-Q water using Slide-A-Lyzer dialysis cassettes (Thermo Fisher Scientific, Japan) with molecular weight cut-off (MWCO) 2000 Da two times for 2 h and one time for 6 h. The solutions of biodots after dialysis were stored in a refrigerator at 4 °C and used for analysis as prepared. Solid samples of biodots for FTIR and elemental analysis were prepared by removing solvent with a rotary evaporator and lyophilization overnight.

### 2.4. Biodots’ Yield Calculation

The yield of biodots (*χ*) was calculated as a ratio of the weight of biodots remained after dialysis, solvent removal, and drying (*m*_biodots_) to the weight of nucleic acid starting material (*m*_nucleic acid_).

$$\chi =\frac{m_{\mathrm{biodots}}}{m_{\mathrm{nucleic}\;\mathrm{acid}}}\times 100\%$$

### 2.5. Quantum Yield (QY) Measurement

The quantum yield of biodots was calculated by comparing the integrated fluorescence intensities against the absorbance values of the samples at 350 nm using quinine sulfate in 0.1 M H_2_SO_4_ as a standard reference. The quantum yield was calculated using the equation below:

$$\varphi_{S}=\varphi_{R}\frac{I_{S}}{I_{R}}\frac{A_{R}}{A_{S}}\left(\frac{n_{S}}{n_{R}}\right)$$

where *φ* is the quantum yield, *I* is the integrated fluorescence intensity, *A* is the absorbance at *λ* = 350 nm, and *n* is the solvent refractive index. *R* and *S* refer to the quinine sulfate reference and to the biodots sample, respectively. The quantum yield of quinine sulfate equal to 54% was used for calculations.

## 3. Results and Discussion

**Figure 1:**
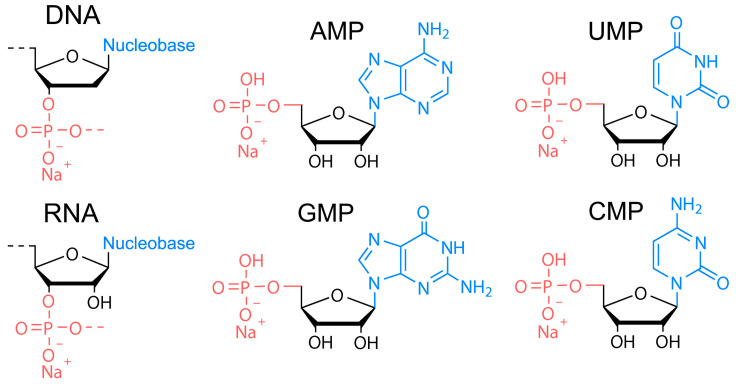
Chemical structures of nucleic acids used as precursors for synthesis of biodots. Chemical structure of DNA, RNA, sodium adenosine monophosphate (AMP), sodium uridine monophosphate (UMP), sodium guanidine monophosphate (GMP), and sodium cytosine monophosphate (CMP).

The DNA of two different molecular weights from salmon sperm (100–300 bp and ca. 20,000 bp), RNA from yeast, and four nucleotides of RNA, that is, sodium adenosine monophosphate (AMP), sodium cytosine monophosphate (CMP), sodium guanidine monophosphate (GMP), and sodium uridine monophosphate (UMP)) were used as precursors (Figure 1) for HT synthesis of biodots. Nucleosides containing no phosphates (adenosine, cytosine, guanidine, and thymidine) were also studied. HT treatment of 1.0% solutions or dispersions of either precursor was performed at varied times and temperatures.

**Figure 2:**
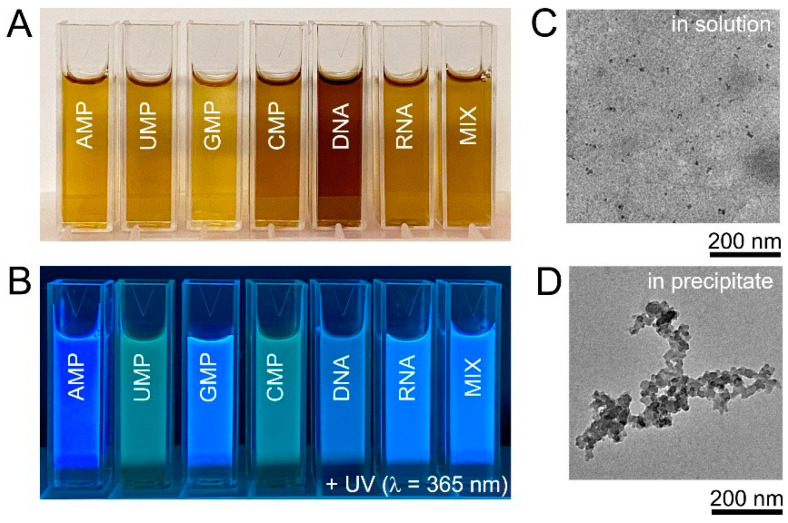
Biodots synthesized from nucleic acids. (**A**). Photographic images of 1% biodot solutions prepared by the HT treatment of AMP, UMP, GMP, CMP, DNA, RNA, and a mixture (MIX) of AMP, UMP, GMP, and CMP nucleotides containing 0.25% of each. (**B**). Corresponding photographs of biodot solutions in the dark under 365 nm UV irradiation. (**C**). Typical TEM image of biodots prepared from DNA by HT treatment. (**D**). Typical TEM image of aggregates in the precipitate formed during DNA HT treatment.

HT treatment at 200 °C for 10 h was chosen as standard synthetic protocol and, unless otherwise mentioned, the results hereinafter were obtained under these conditions. The products of nucleic acid HT treatment were yellow or yellow-brown solutions (Figure 2A). All obtained solutions were strongly fluorescent under UV-A (365 nm) irradiation and, visually, intensities and colours of fluorescent light of obtained products were notably different (Figure 2B). TEM observations of the solutions obtained after the HT treatment of DNA confirmed the presence of small nanoparticles with an average size of 7.8 ± 2.5 nm (Figure 2C), in agreement with the size of DNA biodots reported in the literature that was in the range of 5–20 nm [39,40,41,42]. Samples prepared from DNA and RNA contained black non-fluorescent precipitate (ca. 5%) that was separated by centrifugation. The insoluble precipitate contained carbon particles of several 10 nm in diameter and their aggregates (Figure 2D). It should be noted that the amount of the insoluble precipitate was less than 1% for biodots prepared from nucleotides (Supporting Information, Figure S1).

**Figure 3:**
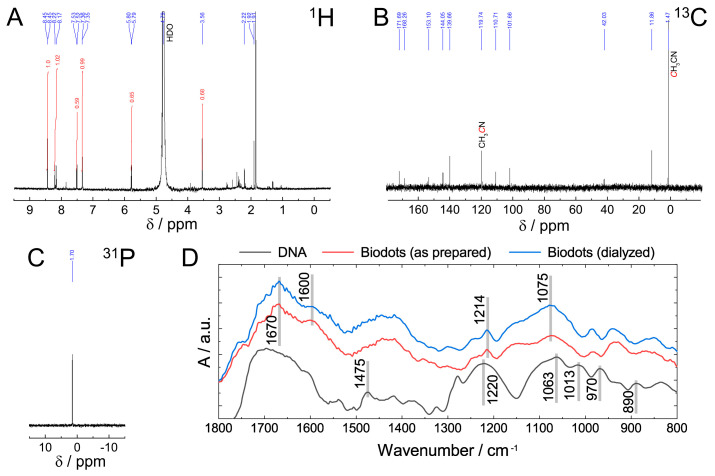
Characterization of biodots prepared from DNA. (**A**–**C**) ^1^H (**A**), ^13^C (**B**), and ^31^P (**C**) NMR spectra of DNA biodots in D_2_O. (**D**). FTIR spectra of DNA, and DNA biodots before and after dialysis.

The chemical structure of DNA nanoparticles (biodots) was assessed by NMR and FTIR spectroscopies (Figure 3). The absence of the characteristic signals of sugar protons in the ^1^H NMR spectrum between 4 and 5 ppm and in ^13^C NMR spectrum between 60 and 90 ppm (Figure 3A,B) indicated a deep degradation of the deoxyribose part of DNA. Most ^13^C NMR signals located between 100 and 170 ppm were assigned to alkene, carbonyl, imine, and aromatic groups (Figure 3B). The signal at 1.7 ppm in ^31^P NMR spectrum (Figure 3C) was assigned to the phosphate remaining from the sugar-phosphate backbone. The position of the signal is different from those reported for DNA or polynucleotides (ca. −4 ppm) [47] and can be assigned to either phosphate ion or monophosphate ester [48]. Considering the fact that the same signal remained after dialysis of biodots, monophosphate ester is considered to be a more likely state than the phosphate ion.

Comparison of FTIR spectra of DNA and DNA-derived nanoparticles (biodots) (Figure 3D) supports the general conclusions of NMR analysis and provides further insight into DNA structural changes during HT treatment. Decomposition of the deoxyribose (sugar) part of DNA is evidenced by the disappearance of bands at 1013 cm^−1^, 970 cm^−1^, and 890 cm^−1^ corresponding to deoxyribose ring vibration (1013 cm^−1^, 890 cm^−1^) and C-C stretching vibration of the ribose-phosphate skeletal backbone (970 cm^−1^) [49]. The absence of characteristic bands of A and G ring vibration (1475 cm^−1^) in biodots indicates that nucleobases undergo substantial transformations. Bands of phosphate moieties corresponding to antisymmetric PO_2_^−^ stretching vibration (1220 cm^−1^) and symmetric PO_2_^−^ stretching vibration (1063 cm^−1^) remain after HT treatment, but shifted to 1214 cm^−1^ and 1075 cm^−1^, respectively. A region between 1600 cm^−1^ and 1700 cm^−1^ has a number of bands corresponding to the stretching vibrations of C=C, C=O, and C=N in biodots.

Elemental analysis (Table 1) was performed to compare chemical composition of DNA biodots (Figure 2C) and the insoluble precipitate (Figure 2D) with that of original DNA to make clear the fate of DNA structural units during HT treatment. Nitrogen content of biodots and precipitate decreased by over two-fold, indicating partial deamination of DNA nucleobases. Carbon content of DNA biodots was much lower than in the original DNA that is explained by decomposition of the deoxyribose moiety, part of which forms a precipitate with a high carbon content. Calculated percentages of O and P indicate notable enrichment of DNA biodots with phosphates in agreement with FTIR and NMR data. In contrast, the percentages of P and O in the precipitate were low. FTIR, NMR, and elemental analysis of AMP biodots showed very similar tendencies to those observed for DNA biodots (Supporting Information, Figures S2 and S3 and Table S1).

**Figure 4:**
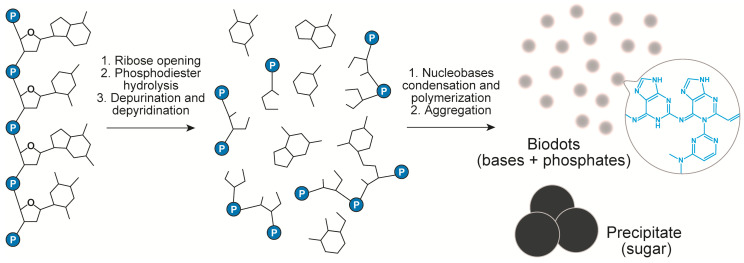
Chemical transformation of DNA during HT treatment.

The above data and the analysis of literature on DNA transformations at high temperatures [50,51,52,53,54,55,56] suggest the following scenario of DNA biodots formation (Figure 4). Heating of DNA solution at temperatures around 100 °C or slightly below results in thermal denaturation of double-stranded DNA to single polynucleotide chains. At the same time, hydrolytic and oxidative damage of DNA leads to deoxyribose ring opening, depurination [53,54] and, to a lesser extent, depyrimidation of DNA single strands, and to partial ring-opening of nucleobases [50]. Nucleobases released in the above process undergo further organic transformations. Condensation of nucleotides through the classical alkylimino-de-oxobisubstitution is considered to be the main transformation scenario [57]. For example, the condensation can occur directly between the carbonyl group of guanine and amino group of cytosine. Secondary amino groups of cytosine can also be involved in the condensation. Furthermore, Uddin et al. [55,56] showed that guanine can be deaminated with the formation of xanthine that can also participate in the condensation reaction. Condensation of nucleobases and products of their transformations having multiple reaction centres (carbonyls and amines) can proceed toward polymerization and crosslinking [57]. The products of such condensation finally assemble into nanoparticles with the help of hydrophobic interactions. Formation of conjugated aromatic systems during condensation gives products with a high fluorescence. Phosphates can either take part in doping of heteroaromatics via the condensation reaction or interact with heteroaromatic condensate electrostatically.

While it was not possible to carry out the comparative HT treatment of pure nucleobases due to their low solubility in water, we performed the HT treatment of D-ribose at 200 °C for 10 h and found that the fluorescent product was not formed (Supporting Information, Figure S4). This confirms that nucleobases play a dominant role in the formation of fluorescent nanoparticles. In good agreement with this scenario, HT treatment of nucleosides containing no phosphates yielded a fluorescent product similar to biodots synthesized from nucleotides (Supporting Information, Figure S5).

**Figure 5:**
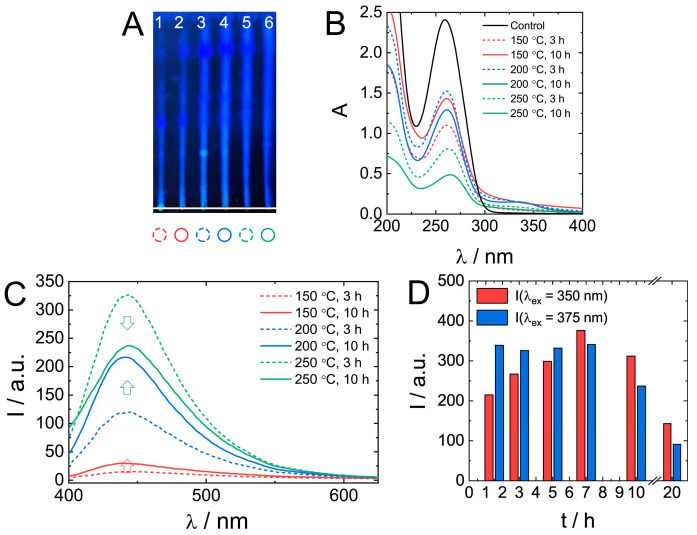
The effect of the HT synthetic conditions on nucleic acids biodots formation and their optical properties. (**A**). A thin layer chromatography of DNA biodots prepared under various treatment times and reaction temperatures (1–150 °C, 3 h; 2–150 °C, 10 h; 3–200 °C, 3 h; 4–200 °C, 10 h; 5–250 °C, 3 h; 6–250 °C, 10 h). A mixture of BuOH:AcOH:H_2_O = 4:1:2 was used as the mobile phase. (**B**,**C**). UV-vis absorbance spectra of 0.02% (*w*/*v*) DNA biodots solutions (**B**) and fluorescence spectra (*λ*_ex_ = 375 nm) of 0.033% (*w*/*v*) DNA biodot solutions (**C**) prepared under various treatment times and reaction temperatures. The control in B is the spectrum of the original DNA solution. (**D**). Dependences of the fluorescence intensity of DNA biodots at *λ*_ex_ = 350 nm and 375 nm on the time of HT treatment at 250 °C.

The effect of synthetic conditions on the properties of biodots was investigated by varying the temperature (150 °C, 200 °C, and 250 °C) and time (3 and 10 h). TLC data (Figure 5A) of as-prepared mixtures indicate the presence of products with different retention characteristics and fluorescence. An increase in HT treatment temperature and time yielded brighter fluorescent products having an increased mobility (larger R_f_) in the silica gel stationary phase.

UV absorbance of original DNA solution decreased by about a half during treatment at 150 °C or 200 °C (Figure 5B). At 250 °C, especially at longer treatment times, the absorbance decreased by as much as 80%. Generally, longer reaction times favoured the formation of a product with higher fluorescence (Figure 5C); however, at 250 °C, shorter reaction times (3 h) resulted in a more fluorescent product. A more detailed analysis of fluorescence dependence on treatment time at 250 °C (Figure 5D) confirmed the decrease of biodot fluorescence intensity at times longer than 10 h. Only ca. 25% of the observed maximal intensity remained after 20 h. Changes in biodot fluorescence at shorter times depended on excitation wavelength, and it was either a gradual increase at *λ*_ex_ = 350 nm or constant fluorescence at *λ*_ex_ = 375 nm. The decrease of fluorescence of a product prepared by a prolonged HT treatment at high temperatures might be due to the decomposition of nucleobases.

**Figure 6:**
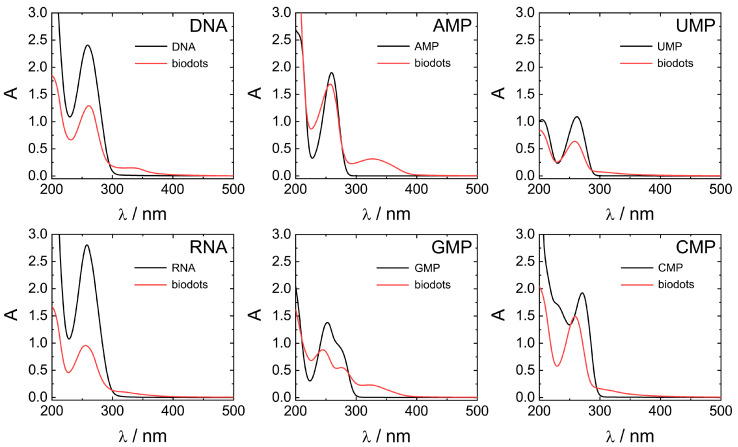
Comparison of UV-vis absorbance properties of nucleic acids and biodots. UV-vis spectra of 0.02% (*w*/*v*) solutions of nucleic acids and the corresponding 0.02% (*w*/*v*) solutions of biodots.

**Figure 7:**
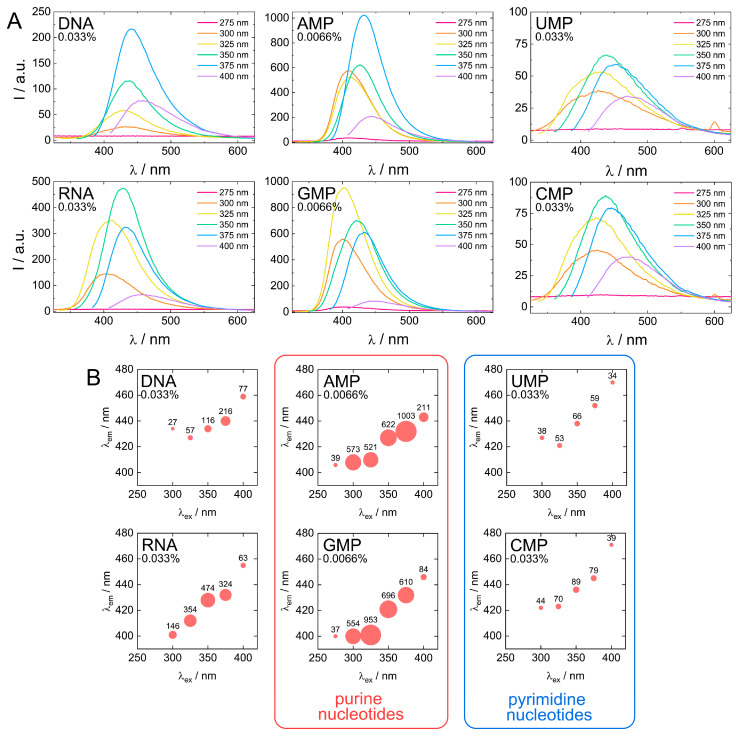
Comparison of fluorescent properties of biodots. (**A**). Fluorescence spectra of 0.033% (*w*/*v*) solutions of biodots prepared from DNA, RNA, UMP, CMP and 0.0066% (*w*/*v*) solutions of biodots prepared from AMP and GMP at different excitation wavelengths *λ*_ex_ = 275–400 nm. Note that concentrations of AMP and GMP used for fluorescence measurements are lower. (**B**). Dependences of maximum fluorescence emission wavelengths (*λ*_em_) and fluorescence intensities of biodots on excitation wavelength (*λ*_ex_). Values of measured fluorescence intensities are shown above the circles in which the areas are proportional to the fluorescence intensities.

Optical properties of biodots prepared from DNA, RNA, and nucleotides were systematically studied by UV-vis and fluorescence spectroscopies (Figure 6 and Figure 7). All nucleic acids are characterized by UV absorbance around *λ* = 260 nm (Figure 6) caused by nucleobases. Generally, HT treatment of nucleic acids resulted in a decrease of the original absorbance peak intensity and, in some cases, appearance of a new absorbance peak or shoulder at ca. 300–350 nm. The decrease of 260 nm peaks was significant for polymeric DNA and RNA, up to 1/3 of original absorbance, and less pronounced for nucleotides. New peaks at 350 nm were more intensive in the case of nucleotides containing purine nucleobases (AMP and GMP) compared to pyrimidine nucleobases (UMP and CMP). Appearance of the peak at 350 nm indicates the transformation of nucleobases into more conjugated structures by condensation of nucleobases is in agreement with the proposed scenario of biodot formation (Figure 4). As we shall see later, the intensity of 350 nm peak is closely related to the fluorescent properties of biodots.

Next, fluorescent characteristics of the biodots prepared from DNA, RNA, and nucleotides were systematically examined and compared (Figure 7A,B). The maximum fluorescence wavelengths (*λ*_em, max_) of all studied biodots increased gradually with an increase of *λ*_ex_ (Figure 7B). The intensity of biodot fluorescence and maximum fluorescence wavelengths were markedly influenced by the structure of biodots’ precursors. Most importantly, we found a large difference between fluorescence intensity of the biodots of the pyrimidine group (UMP and CMP biodots) and purine group (AMP and GMP biodots), which was ca. 50-fold. A very similar correlation was found for biodots prepared from nucleosides (Supporting Information, Figure S3). Quantum yields (QY) of biodots were measured to assess the efficiency of energy transfer (Table 2). Generally, QYs correlated with fluorescence intensities of corresponding biodots (Figure 7). The highest quantum yield of ca. 30% was observed for AMP biodots that is of the same order as the QY of most advanced fluorescent materials prepared from natural sources reported so far [58]. Quantum yields of DNA and RNA biodots were 6.0% and 9.7%, respectively.

Stronger fluorescence of AMP and GMP biodots correlated well with a high UV absorbance at 300–350 nm (Figure 6) in contrast to CMP and UMP. The reason for such a difference can be related to the difference between depurination and depyrimidination kinetics [59]. Faster depurination rates of AMP and GMP make their nucleobase building blocks readily available for condensation into biodots, while the reactions of pyrimidine nucleobases of CMP and UMP are sterically hindered by bound sugar-phosphate moieties. On the other hand, depurination of RNA is known to be more difficult than that of DNA under acidic conditions, yet RNA biodots were more fluorescent (Figure 6 and Table 2). Based on the mechanism suggested above (Figure 4), this indicates that another factor rather than depurination kinetics plays the central role. The difference between DNA and RNA biodots fluorescence can be related to a difference in their nucleobase composition that affects the condensation process and its products.

HT treatment usually yields a complex mixture of nanoparticles coexisting with low-molecular-weight, more fluorescent products [60]. In order to compare properties of an as-prepared product and biodots, the low-molecular-weight fraction was cut off by dialyzing the crude product against a ca. 1 nm pore size membrane (MWCO 2000 Da). Yields of biodots which remained after dialysis are summarized in Table 3. A moderate 20–30% yield of biodots is not only the result of the presence of low-molecular-weight co-products in the product mixture of HT treatment, but is also caused by the loss of the sugar part of the starting material and partial decomposition of nucleobases. Similar yields of biodots prepared from individual nucleotides indicate that the difference in the fluorescence of corresponding biodots shown in Figure 7 is not related to the yield of fluorescent material.

The dialysis resulted in a decrease of biodots solution fluorescence in agreement with past reports [60]. For example, the decrease of DNA HT product fluorescence at *λ*_em_ = 350 nm was about 30%, which is of the same order as the yield of DNA biodots (Table 3). Importantly, FTIR (Figure 3D) as well as ^1^H, ^13^C, and ^31^P NMR spectra of biodots were similar before and after dialysis (Supporting Information, Figure S6). Fluorescent characteristics of biodots before and after dialysis, such as maximum fluorescence wavelengths and relative intensities of biodots from different precursors, remained essentially the same (Supporting Information, Figure S7).

**Figure 8:**
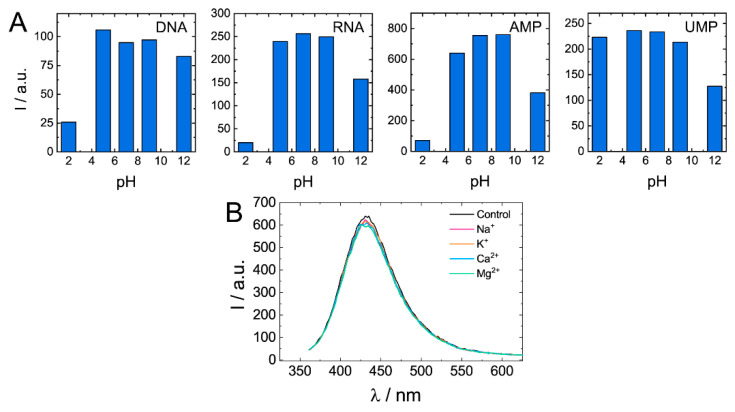
Dependences of biodots fluorescence on pH and the presence of physiologically relevant cations. (**A**). Fluorescence intensities of biodots of DNA, RNA, AMP, and UMP in solutions of various pHs at the excitation wavelength *λ*_ex_ = 375 nm. (**B**). Fluorescence spectra of DNA biodots in solutions containing 0.33 mM of biologically relevant mono- and dications at the excitation wavelength *λ*_ex_ = 375 nm.

Quenching of biodots’ fluorescence was observed under strongly acidic (pH < 3) and strongly alkaline conditions (pH > 11) (Figure 8A). Degree of quenching significantly varied among biodots. For example, while DNA, RNA, and AMP biodots’ fluorescence decreased by ca. 10-fold at pH 2, fluorescence of UMP biodots was the same as at neutral pHs. In the pH range of 5–9, fluorescent characteristics of all studied biodots were not affected by changes in solution pH. Photostability of biodots was also verified in solutions containing physiologically relevant, ubiquitous cations: Na^+^, K^+^, Mg^2+^, and Ca^2+^ (Figure 8B). Neither notable decrease of fluorescence intensity nor the shift of spectra was observed. Good photostability of biodots in solutions of different pH and ionic strengths suggests that they can be used for applications under most physiological and environmental conditions.

**Figure 9:**
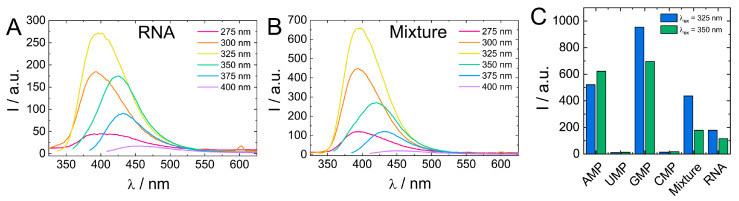
(**A**,**B**). Comparison of fluorescence spectra of 0.01% (*w*/*v*) solutions of biodots prepared from RNA (**A**) and a 1:1:1:1 mixture of AMP:UMP:GMP:CMP (**B**). Concentration of RNA and the total concentration of nucleotides during HT synthesis were the same (1.0%). (**C**). Comparison of fluorescence intensities of biodots prepared from individual nucleotides, their mixture, and RNA at *λ*_ex_ = 325 nm and *λ*_ex_ = 350 nm.

Comparison of biodot fluorescent properties showed that fluorescence of RNA biodots was notably lower compared to the average intensity of biodots prepared from individual RNA nucleosides (Figure 7). Therefore, to gain insight into the effect the polymeric structure of biodots precursor on their fluorescence, we carried out the HT reaction using a 1:1:1:1 mixture of RNA monomers (AMP, UMP, GMP, CMP) of a 1.0% concentration in total and compared the resulting biodots with biodots of RNA (Figure 9).

Biodots prepared from a mixture of RNA nucleotides had a ca. two-fold stronger fluorescence than RNA biodots, while their fluorescent characteristics, that is, fluorescence intensity dependence on excitation wavelength, were basically the same (Figure 9). This difference suggests that the polymeric nature of a precursor has a negative effect on biodot fluorescence. Such a negative effect is related to the mechanism of biodot formation via condensation of nucleobases and can be attributed to steric constrains of nucleobases along the RNA polymer chain, preventing their efficient condensation. To further test this hypothesis, we compared biodots prepared from short (ca. 100–300 bp) and long (ca. 20,000 bp) DNA and found that fluorescence intensity of biodots prepared from shorter DNA was ca. 20% higher (Supporting information, Figure S8) that is in good agreement with the above hypothesis.

Finally, it should be noted that comparison of nucleic acid biodots with fluorescent products prepared from other natural compounds indicates that nucleic acid biodots possess superior fluorescent characteristics. In particular, quantum yields of AMP and GMP biodots are higher than those of biodots prepared from amino acids with an exception of serine [58]. On the other hand, the quantum yield of AMP biodots (30%) is lower than that of classical alloyed semiconductor quantum dots of CdSe and CdTe (ca. 60%) [61], yet biodots have no toxicity and can be prepared from renewable resources that renders them not only more environmentally friendly and sustainable, but also suitable for a broader range of potential applications.

## 4. Conclusions

Fluorescent nanoparticles (biodots) of several nm in size were successfully synthesized from polymeric and monomeric nucleic acids: DNA, RNA, nucleotides, and nucleosides. Formation of biodots proceeds through degradation of ribose sugar, whereas depurination, depyrimidation and consequent condensation and polymerization of nucleobases result in the formation of fluorescent nanoparticles. The chemical structure of precursors strikingly affects the optical properties of fluorescent nanomaterials. In particular, HT treatment of nucleic acids containing purine nucleobases yields biodots that are over 50 times brighter compared to the biodots prepared from precursors containing pyrimidine nucleobases. Biodots prepared from AMP nucleotide have the highest quantum yield of ca. 30%. Individual nucleotides are more suitable for biodot synthesis compared to DNA and RNA macromolecules. The fluorescence of nucleic acid biodots is stable in a broad range of pHs and in the presence of physiologically relevant cations. Further studies on chemical recognition characteristics of biodots prepared from different nucleic acids will address the possibility of using such systems as bioimaging and sensing platforms.

## Figures and Tables

**Table 1:** Elemental analysis of DNA biodots and the insoluble precipitate.

Sample	H/%	C/%	N/%	(O + P) ^a^/%
DNA biodots after dialysis	4.2	21.4	8.1	66.3 ^a^
Precipitate after HT treatment of DNA	4.8	59.0	7.7	28.5 ^a^
Original DNA (theoretical) ^b^	3.6	37.9	17.3	41.2 ^a^

^a^ Calculated as (100% − (H% + C% + N%)); ^b^ Calculated for DNA in form of acid having 50% GC content.

**Table 2:** Quantum yields (QYs) of nucleic acid biodots.

Biodots	AMP	UMP	GMP	CMP	DNA	RNA
QYs/%	29.8	2.0	21.1	3.1	6.0	9.7

**Table 3:** Yields (*χ*) of biodots.

Biodots	AMP	UMP	GMP	CMP	DNA	RNA
*χ*/%	20.3	17.3	17.6	22.0	28.5	18.6

## Supplementary Materials

The following are available online at https://www.mdpi.com/article/10.3390/nano11092265/s1. Figure S1: Analysis of insoluble precipitate fraction after HT synthesis; Figure S2 NMR spectra of AMP biodots; Figure S3: FTIR spectra of AMP and AMP biodots; Figure S4: Fluorescent properties of HT treatment product of ribose; Figure S5: Comparison of fluorescent properties of biodots prepared from nucleosides; Figure S6: NMR spectra of DNA biodots after dialysis; Figure S7: Fluorescent properties of biodots after dialysis, Figure S8: Comparison of biodots prepared from DNA of different molecular weights; Table S1: Elemental analysis of AMP biodots.
