# Supplementary material for: Fluorescent Nanoparticles Synthesized from DNA, RNA, and Nucleotides
Source: Nanomaterials (Basel). 2021 Aug 31;11(9):2265. doi: 10.3390/nano11092265 (PMC8471148; doi:10.3390/nano11092265)
Supplement: Supplementary file 1 [file nanomaterials-11-02265-s001.zip › nanomaterials-1315948-supplementary.pdf]

Supplementary Materials

Fluorescent Nanoparticles Synthesized from DNA, RNA, and Nucleotides

Maofei Wang [1], Masaki Tsukamoto [2], Vladimir G. Sergeyev [3] and Anatoly Zinchenko [1,*]

[1] Graduate School of Environmental Studies, Nagoya University, Furo-cho, Chikusa-ku, Nagoya 464-8601, Japan; wang.maofei@b.mbox.nagoya-u.ac.jp
[2] Graduate School of Informatics, Nagoya University, Furo-cho, Chikusa-ku, Nagoya 464-8601, Japan; tsukamoto@i.nagoya-u.ac.jp
[3] Department of Chemistry, M.V. Lomonosov Moscow State University, Moscow 119899, Russia; sergeyevvg@gmail.com
* Correspondence: zinchenko@urban.env.nagoya-u.ac.jp; Tel.: +81-52-789-4771

1. Insoluble Precipitate Fraction after Hydrothermal Synthesis

The amount of the insoluble precipitate was ca. 5% for DNA, RNA, and GMP, and less than 1% for AMP, UMP, and CMP.

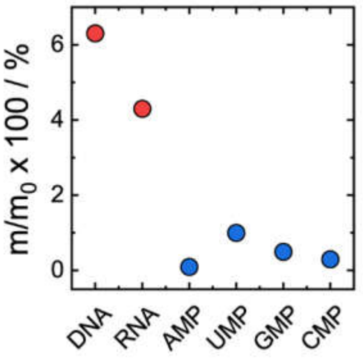

Figure S1. A fraction (%) of insoluble precipitate separated by centrifuging at 4000 rpm for 10 min after HT treatment of nucleic acid precursors at 200 °C for 10 h.

2. NMR Spectra of AMP Biodots before Dialysis

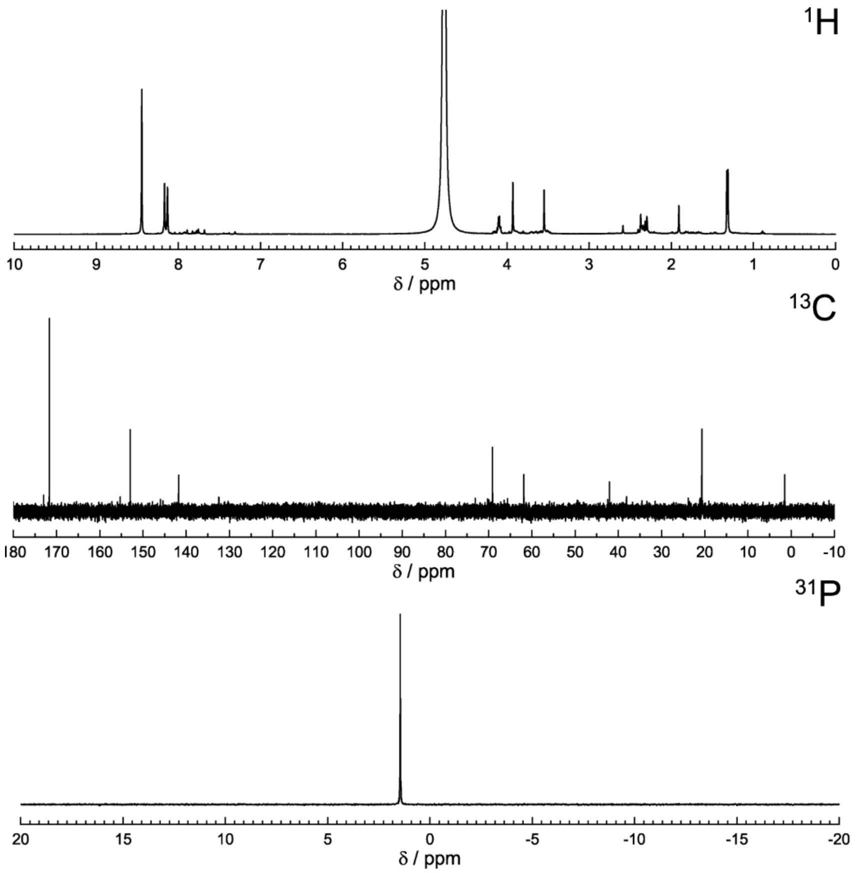

Figure S2. ^{1}H (A), ^{13}C (B), and ^{31}P (C) NMR spectra of AMP biodots in D_2O after dialysis.

3. FTIR Spectra of AMP and AMP Biodots

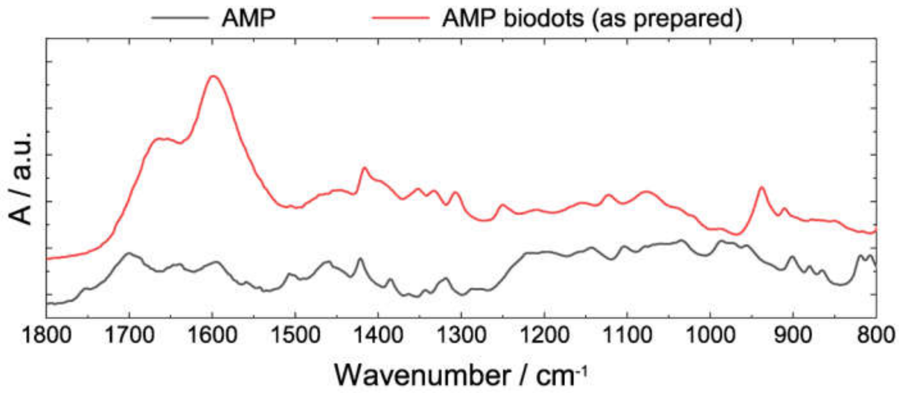

Figure S3. FTIR spectra of AMP and AMP biodots as prepared.

4. Hydrothermal Treatment of Ribose

No fluorescent product was formed after the hydrothermal treatment of ribose at 200 °C for 10 hours.

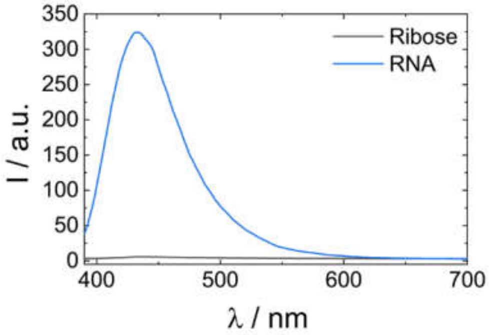

Figure S4. Comparison of the fluorescent spectra of fluorescent products prepared from RNA and D-(–)-ribose by a hydrothermal treatment at 200 °C for 10 hours. The excitation wavelength was λ_{ex} = 375 nm.

5. Comparison of Fluorescent Properties of Biodots Prepared from Nucleosides

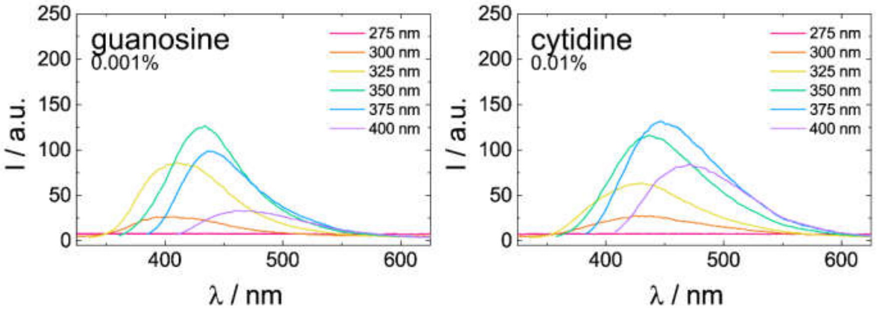

Figure S5. Fluorescence spectra of 0.001% (w/v) solution of biodots prepared from guanosine and 0.01% solution biodots prepared by a hydrothermal treatment at 200 °C for 10 hours from cytidine at different excitation wavelength λ_{ex} = 275–400 nm. Note that concertation of guanosine and cytidine are different.

6. NMR Spectra of DNA Biodots after Dialysis

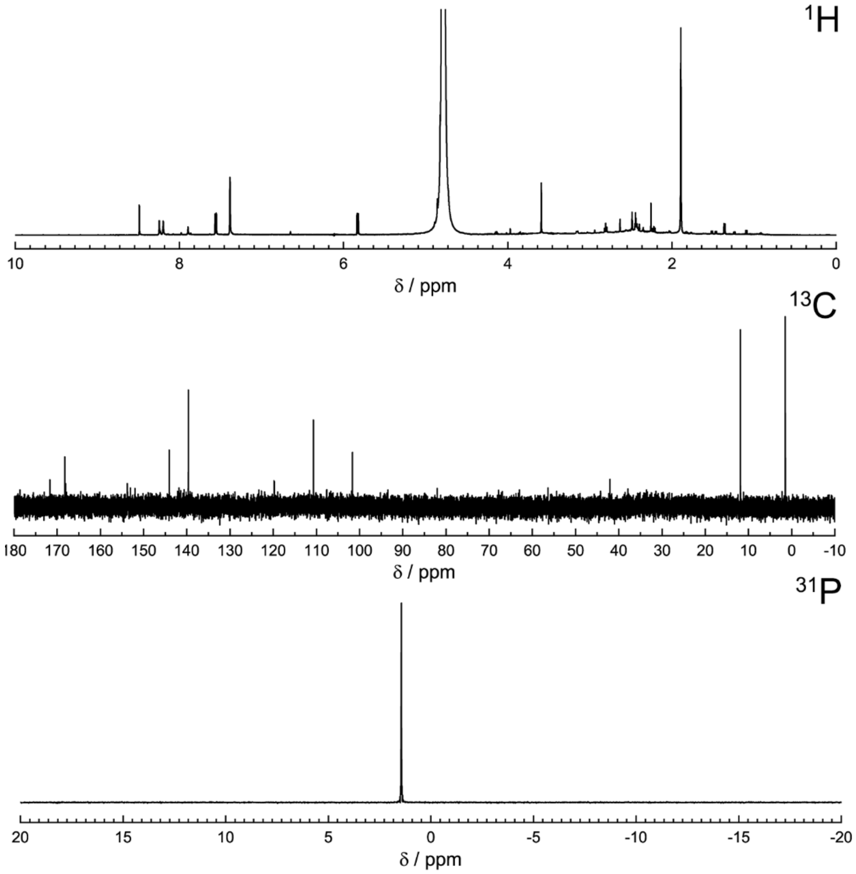

Figure S6. ^{1}H (A), ^{13}C (B), and ^{31}P (C) NMR spectra of DNA biodots in D_2O after dialysis.

7. Fluorescent Properties of Biodots after Dialysis

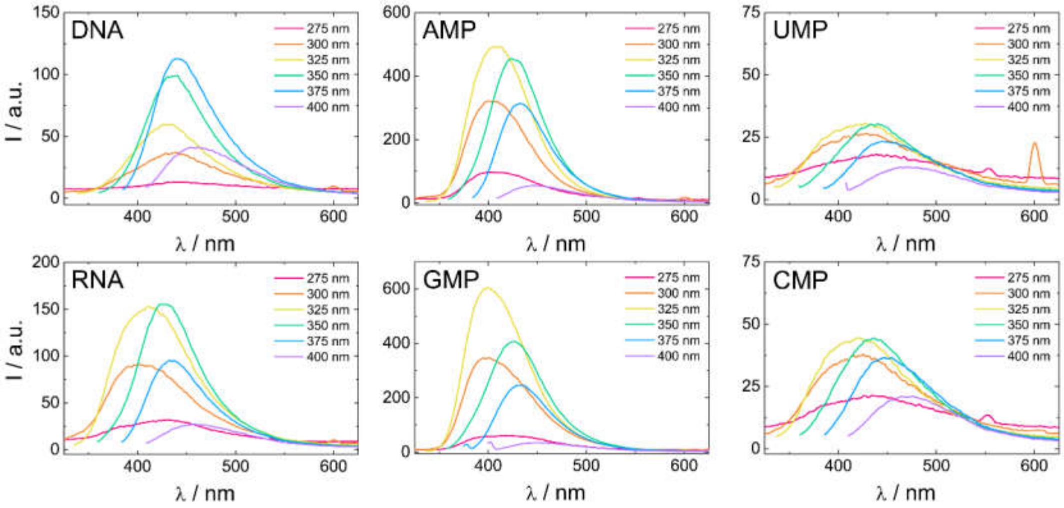

Figure S7. Fluorescence spectra of biodots solutions prepared from DNA, RNA, and nucleotides after dialysis using a dialysis membrane with molecular weight cutoff 2000 Da.

8. Comparison of Fluorescent Spectra of Biodots Prepared from DNA of Different Molecular Weight

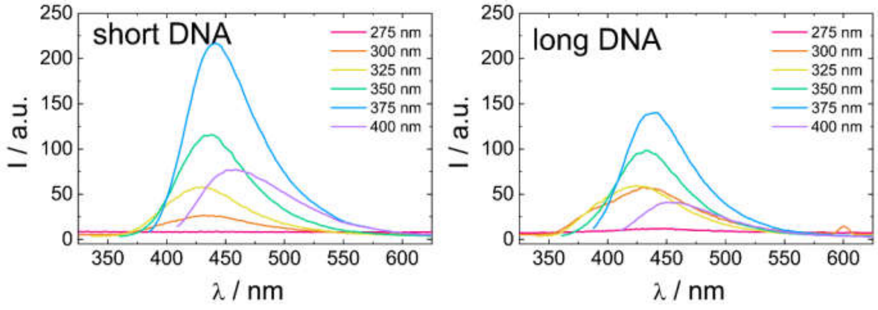

Figure S8. Comparison of fluorescent spectra of 0.01% (w/v) solutions of biodots prepared from high (> 20,000 bp) and low (*ca.* 100–300 bp) molecular weight DNA. Concertation of either DNA precursor during HT treatment was 1%.

Table S1. Elemental analysis of AMP biodots.

Sample	H / %	C / %	N /%	(O + P)[a] / %
AMP biodots after dialysis	5.19	20.99	11.51	62.32
Original AMP (theoretical)	4.06	34.59	20.17	41.18

[a] Calculated as (100% – H% – C% – N%).
